# Understanding and looking after a retinoscope and trial lens set

**Published:** 2017-08-07

**Authors:** Ismael Cordero

**Affiliations:** 1 Clinical Engineer, Philadelphia, USA.

**A well-cared-for retinoscope and trial lens set, for the measurement and correction of refractive errors, will give many years of service.**

Retinoscopy is the use of a retinoscope to measure a patient's refractive error. Retinoscopy is an objective method of refraction in which the patient does not need to tell the practitioner how they see. If instead they ask the patient questions about how she/he sees, that is called subjective refraction.

When the practitioner shines the light of a retinoscope into an eye, they see the light reflected from the retina. This reflected light is called the retinoscopic reflex, or ‘ret reflex’; it looks like a red light inside the pupil. Depending on the person's refractive error, when the practitioner moves the retinoscope, the ret reflex will move in a certain way inside the pupil. Trial lenses can be used to measure the amount of movement that a ret reflex has, so that the refractive error can be estimated accurately.

There are two types of retinoscopes:

1. **Streak retinoscopes** have a light source that produces a line or streak of light. The streak of light can be changed by moving the slide knob or sleeve (Figure 1). It can be:
  - rotated to any axis position (by rotating the sleeve)
  - made wider or narrower in width (by moving sleeve up or down)
  - changed from convergent to divergent light (by moving the sleeve up or down). It is normally used in the ‘down’ position.
2. **Spot retinoscopes** have a light source that produces a spot of light. The spot of light can be changed by moving its slide knob. It can be:
  - made larger or smaller in diameter (by moving the sleeve up or down)
  - changed from convergent to divergent light (by moving the sleeve up or down).

The spot light of a spot retinoscope does not need to be rotated (like the streak retinoscope) to examine different axis directions.

The ret reflex can be neutralised with plus and minus trial lenses:

- Plus lenses neutralise a ‘with’ movement.
- Minus lenses neutralise an ‘against’ movement.

A trial lens set can include up to 266 lenses:

- Spherical lenses, with a wide range of powers, both positive and negative, generally 0.12 D, 0.25 D, and then in steps of 0.25 D up to a certain point, then in steps of 0.50 D, and then finally in steps of 1.00 D. At least two of each power are included.
- Cylindrical lenses in a variety of powers, often both positive and negative, mostly in steps of 0.25 D.
- Accessory lenses used for special tests: prisms, filters, occluders, pinhole, and others.

**Figure 1:**
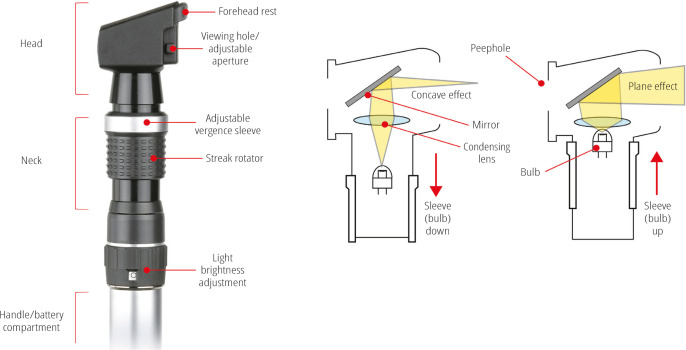
Retinoscope components: streak retinoscope

**Figure 2:**
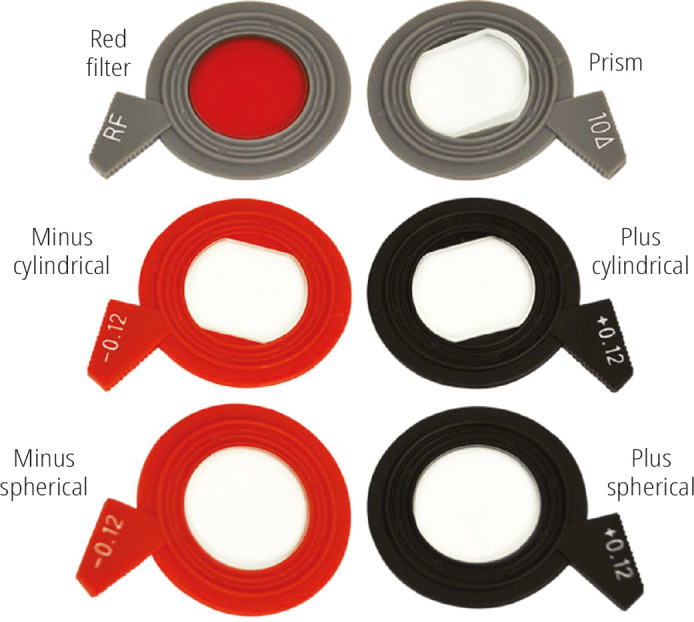
Typical trial lenses

Figure 2 shows some typical trial lenses.

A trial lens set also includes a trial frame which is needed to hold the lenses over each eye. A typical frame can be seen in Figure 3.

The positions of the two side assemblies can be adjusted laterally by two small knobs. The positions can be read on scales. The sum of the two readings is the interpupillary distance (PD).

The nosepiece assembly can be adjusted in two ways. It can be moved fore-and-aft, to adjust the distance of the trial lenses from the eye to meet the standard distance or the measured distance chosen for the patient. It can be adjusted up-and-down, to place the centres of the lenses vertically in line with the patient's pupils.

The temple pieces have adjusting knobs that adjust their angle with respect to the frame. The lengths of the temple pieces can also be adjusted to the location of the patient's ears after the eye distance has been set.

On each side, there is a ring with two bars, each having three cells to receive trial lenses, plus a set of three spring clips for holding them in place.

Each ring can be separately rotated by means of a small, knurled knob. A scale gives the orientation of the axis of a cylinder lens, indicated by a dot or straight line marking on the lens.

## General care

### Retinoscope

Wipe the external surface of the retinoscope with a cloth dampened with a mild detergent and water solution, or a 70% isopropyl alcohol solution, or a 10% bleach solution (by volume); however, always consult the manufacturer's user manual.

When wiping, avoid the optical surfaces.

**NOTE:** solution entering the assembly could damage internal components. Use caution to ensure cloth is not saturated with solution.

Wipe the lenses with a dry lens-cleaning cloth.

Periodically check the following - the answer for all the below should be yes:

- Do you get a streak of uniform brightness when the instrument is turned on?
- Does the brightness of the streak vary when the brightness control is turned?
- Does the sleeve in the instrument slide up and down freely and vary the length when it is moved?
- Does the patch of light or the streak rotate when the bulb is turned using the sleeve?

### Trial lens set

- Wipe lenses with a lens cleaning cloth if stained or smudged.
- Keep the lenses in the case after use.
- Wipe the trial frame's nose rest with an alcohol wipe after each patient.

**Figure 3:**
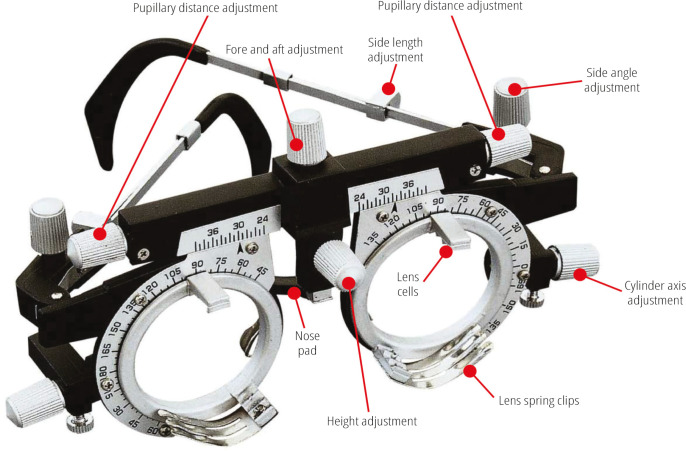
Trial frame components

